# Impact of drug and equipment preparation on pre-hospital emergency Anaesthesia (PHEA) procedural time, error rate and cognitive load

**DOI:** 10.1186/s13049-018-0549-3

**Published:** 2018-09-21

**Authors:** Paul Swinton, Alasdair R. Corfield, Chris Moultrie, David Percival, Jeffrey Proctor, Neil Sinclair, Zane B. Perkins

**Affiliations:** 1 Emergency Medical Retrieval Service, ScotSTAR, Scottish Ambulance Service, Glasgow, UK; 2 Scottish Air Ambulance Division, Scottish Ambulance Service, Glasgow, UK; 3 Royal Alexandra Hospital, Paisley, UK; 4 ScotSTAR, Scottish Ambulance Service, Glasgow, UK; 5 Scottish Ambulance Service, Clinical Directorate, Edinburgh, UK; 6 Centre for Trauma Sciences, Queen Mary, University of London, London, UK

**Keywords:** Airway, Intubation, Emergency, Patient safety / safety, Human error, Drug preparation, Risk management, Human factors

## Abstract

**Background:**

We examined the effect of advanced preparation and organisation of equipment and drugs for Pre-hospital Emergency Anaesthesia (PHEA) and tracheal intubation on procedural time, error rates, and cognitive load.

**Methods:**

This study was a randomised, controlled experiment with a crossover design. Clinical teams (physician and paramedic) from the Emergency Medical Retrieval Service and the Scottish Air Ambulance Division were randomised to perform a standardised pre-hospital clinical simulation using either unprepared (standard practice) or pre-prepared (experimental method) PHEA equipment and drugs. Following a two-week washout period, each team performed the corresponding simulation. The primary outcome was intervention time. Secondary outcomes were safety-related incidents and errors, and degree of cognitive load.

**Results:**

In total 23 experiments were completed, 12 using experimental method and 11 using standard practice. Time required to perform PHEA using the experimental method was significantly shorter than with standard practice (11,45 versus 20:59) minutes: seconds; *p* = < 0.001). The experimental method also significantly reduced procedural errors (0 versus 9, *p* = 0.007) and the cognitive load experienced by the intubator assistant (41.9 versus 68.7 mm, *p* = 0.006).

**Conclusions:**

Pre-preparation of PHEA equipment and drugs resulted in safer performance of PHEA and has the potential to reduce on-scene time by up to a third.

**Electronic supplementary material:**

The online version of this article (10.1186/s13049-018-0549-3) contains supplementary material, which is available to authorized users.

## Background

The primary purpose of an ambulance service is to provide rapid access to emergency care. This involves prompt, effective pre-hospital care and rapid transport to hospital.

Pre-hospital interventions that delay transport to hospital may worsen outcome [1]. For effective pre-hospital care, it is therefore important that the likely benefit of any intervention is weighed up against potential risks, including delayed transport to hospital.

Pre-hospital Emergency Anaesthesia (PHEA) with oral tracheal intubation is the technique of choice to manage critically ill or injured patients who cannot maintain their airway or achieve adequate ventilation [2]. While a potentially life-saving intervention in this group of patients, PHEA is associated with significant risks and is a recognised cause of prolonged on-scene times [1–5]. Most PHEA complications are predictable, and risk can be significantly reduced with appropriate preparation [2]. However, it is this preparation step that accounts for the majority of procedural time. A particular challenge in improving the overall benefit of PHEA is to reduce the time penalty of the procedure while ensuring that the highest safety standards are achieved [2].

In the UK, thirty pre-hospital services provide PHEA, and perform approximately 1600 PHEA procedures a year [6]. There is some variability between services with regard to the amount of PHEA preparation that is done prior to tasking, and the amount done on-scene. The busiest services pre-prepare equipment and drugs prior to tasking, while the majority of services perform this step on-scene.

The aim of this study was to evaluate the effect of pre-prepared equipment and drugs, on PHEA procedure time and safety. In addition, we assessed the effect that pre-preparation had on the cognitive load of clinicians. We hypothesised that the use of pre-prepared PHEA equipment and drugs could reduce procedural time and risk, thus improving the overall benefit of the intervention.

## Methods

### Study design

**Fig. 1:**
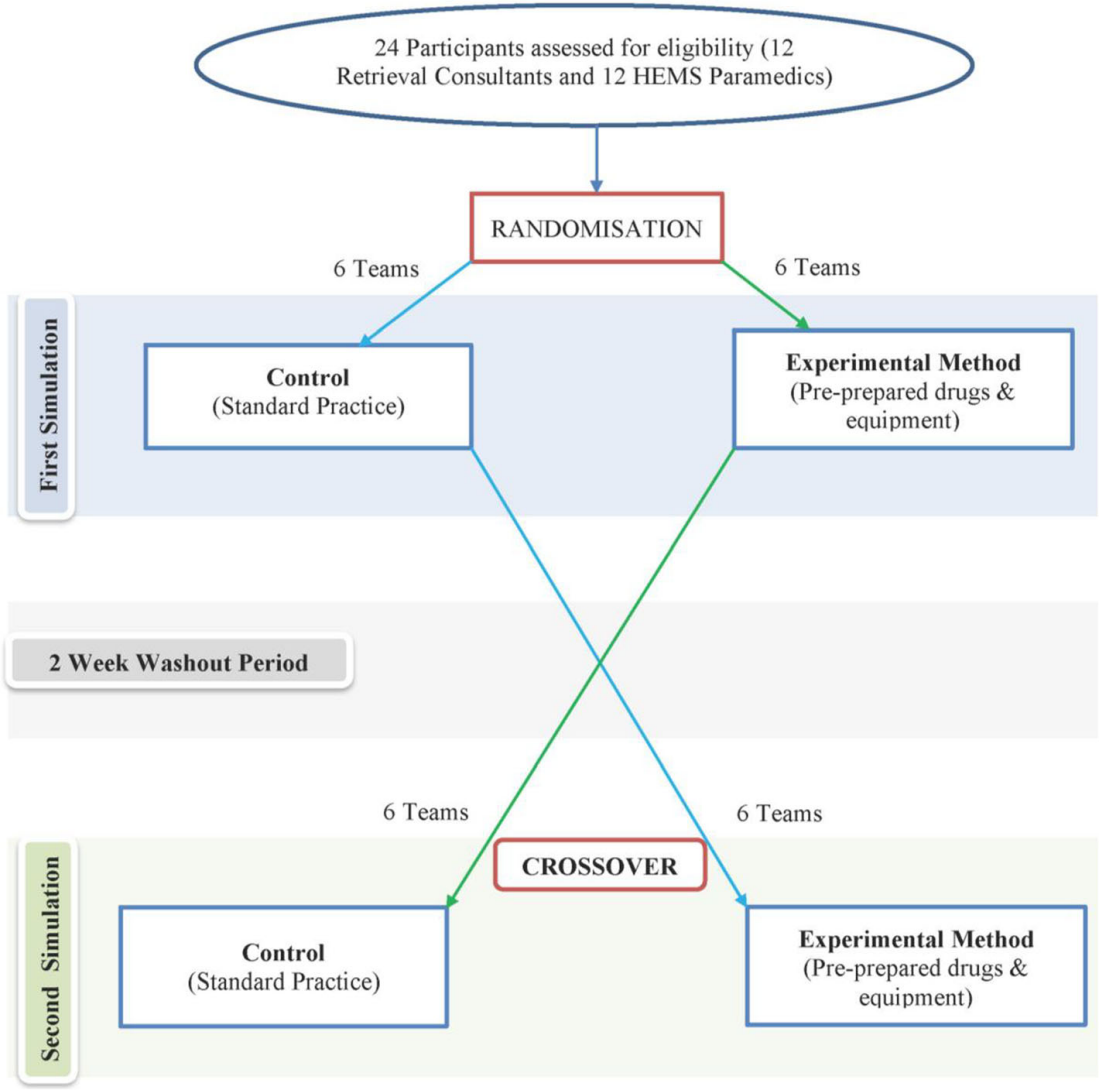
Study Scheme Diagram

This was a randomised, controlled simulation experiment with a crossover design. The study design is presented in Fig. 1 with the full study protocol showing more detail [see Additional file 1].

Experiments were conducted between 04 January and 01 March 2017. The study was reviewed and approved by Queen Mary, University of London Research Ethics Committee (QMREC1839a), Greater Glasgow and Clyde Clinical Research & Development committee (GN16AE762), and the Scottish Ambulance Service. Written informed consent was obtained from participants.

### Participants and setting

The study was undertaken at Scotland’s national Specialist Transport and Retrieval service (ScotSTAR). The service exists to provide a safe and dedicated transport and retrieval service to the 5.5 million population of Scotland [7, 8].

The Emergency Medical Retrieval Service (EMRS), ScotSTAR’s adult retrieval service is a physician-led team delivering a primary pre-hospital response, working with the ambulance service to provide pre-hospital critical care at the scene of incidents. The EMRS team comprises a consultant physician, and a retrieval practitioner. When deployed by air, the EMRS team delivers PHEA with a Helicopter Emergency Services (HEMS) Paramedic in the role of intubator assistant.

The service has strict clinical governance procedures, which includes an intense training program prior to independent pre-hospital practice; adherence to SOPs that govern all aspects of pre-hospital practice, including the delivery of PHEA; and regular simulation training in the application of these procedures.

Eligible participants are experts in pre-hospital care, and perform PHEA as part of their normal working practice. Consultant retrieval physicians were recruited from the Emergency Medical Retrieval Service (EMRS), ScotSTAR’s adult retrieval service. As this study was conducted within the Greater Glasgow and Clyde NHS board (NHS GG&C) only consultant physicians currently working for the Emergency Medical Retrieval Service and employed by NHS GG&C were permitted to participate.

Paramedics were recruited from the Scottish Ambulance Service pool of HEMS Paramedics.

Consultant retrieval physicians were eligible for enrolment if they were: 1) currently working with EMRS and employed by NHS GG&C, 2) had > 6 months’ experience as a retrieval physician, and 3) had been assessed by EMRS to be competent and current at PHEA.

HEMS paramedics were eligible for enrolment if they 1) were currently working for the SAS air ambulance division alongside EMRS, 2) had > 6 months’ experience as a retrieval paramedic, 3) had > 8 years’ experience as a paramedic, 4) had completed a recognised PHEA course [9], and 5) were assessed by EMRS to be competent and current at assisting PHEA.

All eligible participants were emailed an invitation to participate in the study. Willing participants were randomized using a computerised random number generator to identify which eligible participants would be enrolled into the study*.*

### Randomisation and allocation concealment

A third party, not involved with enrolment and unaware of the study outcomes, used a computerised random number generator to block randomised participants into 12 two-person (physician /paramedic) teams. In addition, computer batch randomisation was used to establish the first simulation that would be undertaken by the team (standard practice or experimental method). Clinical teams were randomised to either the standard practice or experimental arm. Following a two-week wash-out period, the same clinical teams performed the corresponding simulation. Before each simulation, teams received a standardised briefing, including review of the services standard operating procedure (SOP) [10] and opportunity to prepare and ask questions. Teams were blind to outcomes being measured.

### Interventions

In both arms of the experiment, the clinical team performed PHEA on a mannequin, presented within a realistic pre-hospital clinical simulation [Additional file 2]. This included the decision to perform PHEA, and the performance of the procedure according to the services SOP. The preparation for PHEA involves establishing an equipment “kit dump” as well as the preparation and administration of drugs (Alfentanil (1 mg intravenously (IV)), Ketamine (2 mg/kg IV) and Rocuronium (1–1.2 mg/kg IV)). Correct placement of the tracheal tube (ETT) is confirmed by visualising it pass the vocal cords, by auscultation and by the measure of quantitative end-tidal capnography (EtCO_2_), before securing it in place.

The fidelity of the simulation required it to be performed accurately in accordance with the ScotSTAR PHEA protocol [10], safely, in real time and with the retrieval consultant physician undertaking the role of intubator, and the HEMS paramedic the role of assistant.

**Fig. 2:**
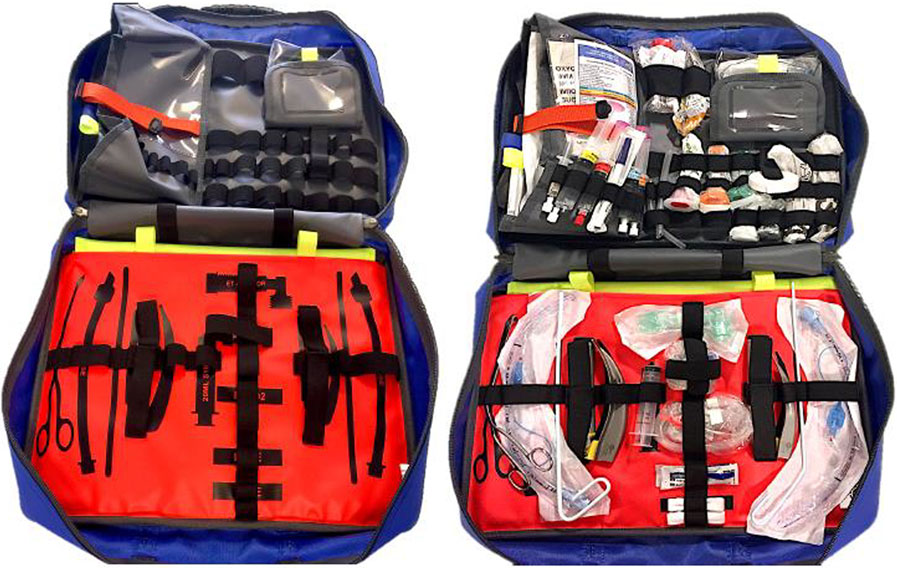
The Adult SCRAM Bag. “The Adult SCRAM (Structured CRitical Airway Management) Bag is an Emergency Airway Bag which provides a structured reproduceable approach to airway management”[34].

**Fig. 3:**
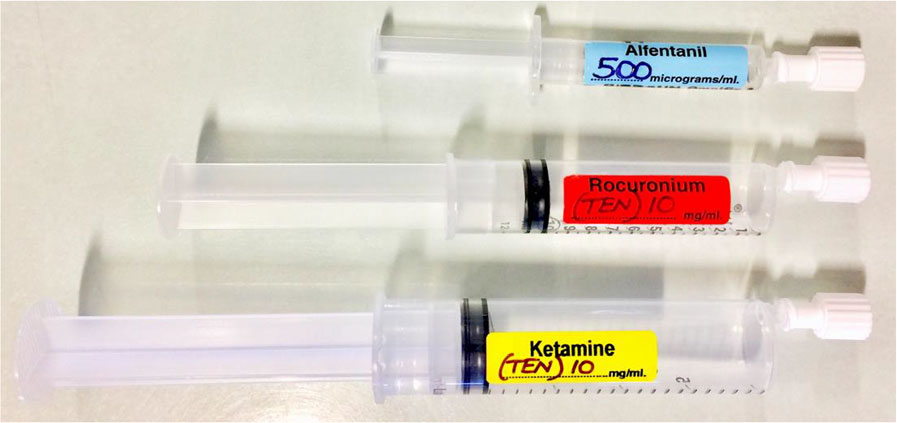
Pre-prepared anaesthesia medications. Drugs are prepared in syringes that are clearly labelled with the agents name and concentration.

The standard practice arm (unprepared), consisted of a drug bag containing all the required drug vials, syringes and labels to prepare for a PHEA, and a conventional airway bag holding all the required airway equipment. In this method, the “kit dump” and drugs are prepared according to the SOP after the decision to intubate has been made. The experimental method consisted of, equipment and drugs, optimally organised and prepared prior to the procedure being required (pre-prepared), having the kit dump pre-prepared within the airway bag, with individual items held securely in place (Fig. 2), and the drugs pre-prepared in labelled syringes (Fig. 3).

### Methods of measurements and outcomes measures

Simulations were filmed, which allowed accurate measurement of outcomes and maintained blinding of participants to the outcomes being measured. Two reviewers independently analysed recordings and extracted study data from the video recording into a pre-prepared spreadsheet. Any discrepancy was resolved by consensus with a third independent reviewer who was blind to the study aims*.*

A full pre-hospital clinical simulation was simulated to reduce moulage artefact and mask the aspects of pre-hospital care that were being measured. Participants were blind to the study outcomes measured during the simulation (intervention time and error rate). Primary outcome was PHEA intervention time (minutes: seconds). Intervention time was defined as starting at the decision to perform PHEA and ending when correct ETT position was confirmed with the facilitator turning on the EtCO_2_ simulation software, in response to visualising chest inflation. Secondary outcomes included procedural errors, defined as an unintended/unexpected incident, which led, or could have led to harm. Errors were counted and classified according to Table 1. In addition, we assessed the degree of individual cognitive load (ICL), defined as the amount of cognitive work/energy required by the participant to complete the procedure, including the level of judgements/decisions needing to be made. ICL was measured using Visual analogue score (VAS). At the end of each simulation, participants were asked to indicate the magnitude of their perceived cognitive load during PHEA by marking a standard 100-mm line appropriately (0-mm representing no cognitive load and 100-mm representing maximal cognitive load). Visual analogue scores have been shown to be effective tools for measuring cognitive load [11, 12].

**Table 1:** Error Classifications

Classification	Definitions	Examples
Error	Procedural error in the preparation or use of medications or equipment with the potential to result in harm.	**Medication**: ○ Syringe containing anaesthesia medication labelled incorrectly or not labelled ○ Incorrect medication administered ○ Incorrect dose administered **Equipment:** ○ Sharps injury ○ Procedure performed not in accordance with SOP (i.e. checklist not used, bougie not used)
Lapse	A failure to execute an action due to lapse in memory and a routine behaviour being omitted.	**Medication preparation:** ○ Same needle used to draw up multiple medications ○ No syringe cap ○ Unsafe sharps management **Equipment preparation:** ○ Cuff of tracheal tube not checked ○ Laryngoscope bulb operation not checked ○ No bougie

### Statistical analysis

Statistical analysis was performed using SPSS 24.0 software (SPSS Inc., Chicago, IL, USA). Shapiro-Wilk test and normal-quartile plots were used to assess normality. Categorical data are reported as frequency (n) and percent (%), and numerical data are reported as mean with Standard Deviation (SD) or median with Interquartile Range (IQR). Sample size was calculated using data from observation of prior EMRS practice: in ten consecutive standard PHEA’s, the mean procedural time was 20:03 (3:26) minutes: seconds. A 20% reduction in procedural time was considered clinically significant. We determined that eleven simulations in each arm of the study were required for the paired t-test to have a 90% chance of detecting a difference in means of four minutes at a level of significance of 5% (two-sided). We adjusted the sample size to twelve simulations in each arm to allow for any exclusions.

Parametric data were compared using the paired *t*-test and non-parametric data were compared using Wilcoxon matched-pair signed-rank test. An absolute (Mean Difference, MD, or Difference of Medians, DM) measure of intervention effect with 95% Confidence Intervals (CI) was calculated for primary and secondary outcomes. A two-tailed *P*-value < 0.05 was considered significant.

## Results

### Characteristics of study participants

Twenty-three simulations (11 in standard arm, 12 in experimental arm) were completed and the data included in this analysis. Characteristics of participating clinicians are described in Table 2. One simulation (standard arm) could not be completed due to operational demands. The outcomes of PHEA simulation using the two methods of equipment and drug preparation are presented in Table 3.

**Table 2:** Baseline Characteristics of study participants

Characteristics	Consultant Physicians (*n* = 12)	HEMS Paramedics (n = 12)
Age (years)	43 (34 to 53)	44 (35 to 49)
Gender (male)	12 (100)	9 (75)
Background Speciality		
Emergency Medicine	10 (83)	n/a
Anaesthetists / Intensivist	2 (17)	n/a
HEMS Paramedic	n/a	12 (100)
NHS Consultant Physician Experience (years)	9 (2 to 16)	n/a
Paramedic Experience (years)*	*n/a*	13.5 (8 to 28)
PHEA Experience (years)	9 (4 to 14)	2.5 (0.5 to 7)

*Median experience of HEMS paramedics as frontline ambulance paramedics

**Table 3:** Outcomes - PHEA simulation using standard practice and an experimental method of equipment and drug preparation

Outcome	Standard Practice (11 simulations)	Experimental Method (12 simulations)	Mean Difference (95% CI)	*P*-value
Intervention Time (min:sec)	20:59 (3:13)	11:45 (1:45)	9:14 (7:42 to 10:45)	< 0.001
Errors	9 (0 to 17)	0 (0 to 2)	8.6 (4.5 to 12.8) *	0.007
Cognitive Load Intubator (mm)	49.9 (20.5)	49.4 (20.5)	0.5 (−16.7 to 17.8)	0.945
Cognitive Load Assistant (mm)	68.7 (24.8)	41.9 (22.4)	26.8 (9.8 to 43.1)	0.006

* *Difference of Medians with 95% CI*

### Primary outcome: Intervention time

Overall, the average intervention time was 16:13 (SD 5:17) minutes: seconds. Teams were able to perform PHEA significantly faster using the experimental method compared to standard practice (11:45 (SD 1:45) versus 20:59 (SD 3:13); MD 9:14 (95% CI, 7:42 to 10:45) minutes: seconds; p = < 0.001) (Students *t*).

Group comparison demonstrated that slow working teams worked slowly in both methods, and faster working teams worked faster in both methods. Despite this, all teams were significantly quicker when using the experimental method. Pre-preparation of drugs resulted in the largest procedural time savings (Table 4).

**Table 4:** Component Times

Component (min:sec)	Standard Practice (11 simulations)	Experimental Method (12 simulations)	Mean Difference (95% CI)	*P*-value
Equipment preparation	05:01 (03:42 to 06:19)	03:57 (03:13 to 04:41)	01:04 (− 00:18 to 02:26)	0.114
Drug Preparation	08:23 (06:43 to 10:03)	00:16 (00:07 to 00:24)	08:07 (06:26 to 09:49)	< 0.001
Checklist	03:50 (03:02 to 04:38)	03:40 (03:12 to 04:08)	00:10 (−00:27 to 00:47)	0.551
Drug Administration Including Onset Time	01:36 (01:16 to 01:57)	01:40 (01:31 to 01:49)	−00:04 (− 00:24 to 00:17)	0.702
Tracheal Intubation	00:42 (00:32 to 00:51)	00:37 (00:32 to 00:43)	00:04 (−00:07 to 00:15)	0.407
Total Intervention time	20:59 (18:49 to 23:09)	11:45 (10:34 to 12:56)	09:14 (07:42 to 10:45)	< 0.001

Data presented as mean (95% CI)

Definitions of Pre-hospital Anaesthesia components [see Additional file 3]

### Secondary outcomes

#### Procedural errors

Overall, 99 errors occurred during the 23 PHEA simulations (Table 5). Significantly fewer errors occurred when teams used the experimental method compared to standard practice: (0 (IQR: 0 to 2) versus 9 (IQR: 0 to 17); DM 9 (95% CI, 4.5 to 12.8); *p* = 0.007) (Wilcoxon matched-pair signed-rank).

**Table 5:** Characteristics of procedural errors

Characteristics	Standard Practice	Experimental Method	*p*-value
Laps in medication preparation	41	0	0.007
Error in medication preparation	31	0	0.011
Laps in equipment preparation	23	2	0.0027
Error in equipment preparation	2	0	0.317
Total errors	97	2	0.007

#### Cognitive load

**Fig. 4:**
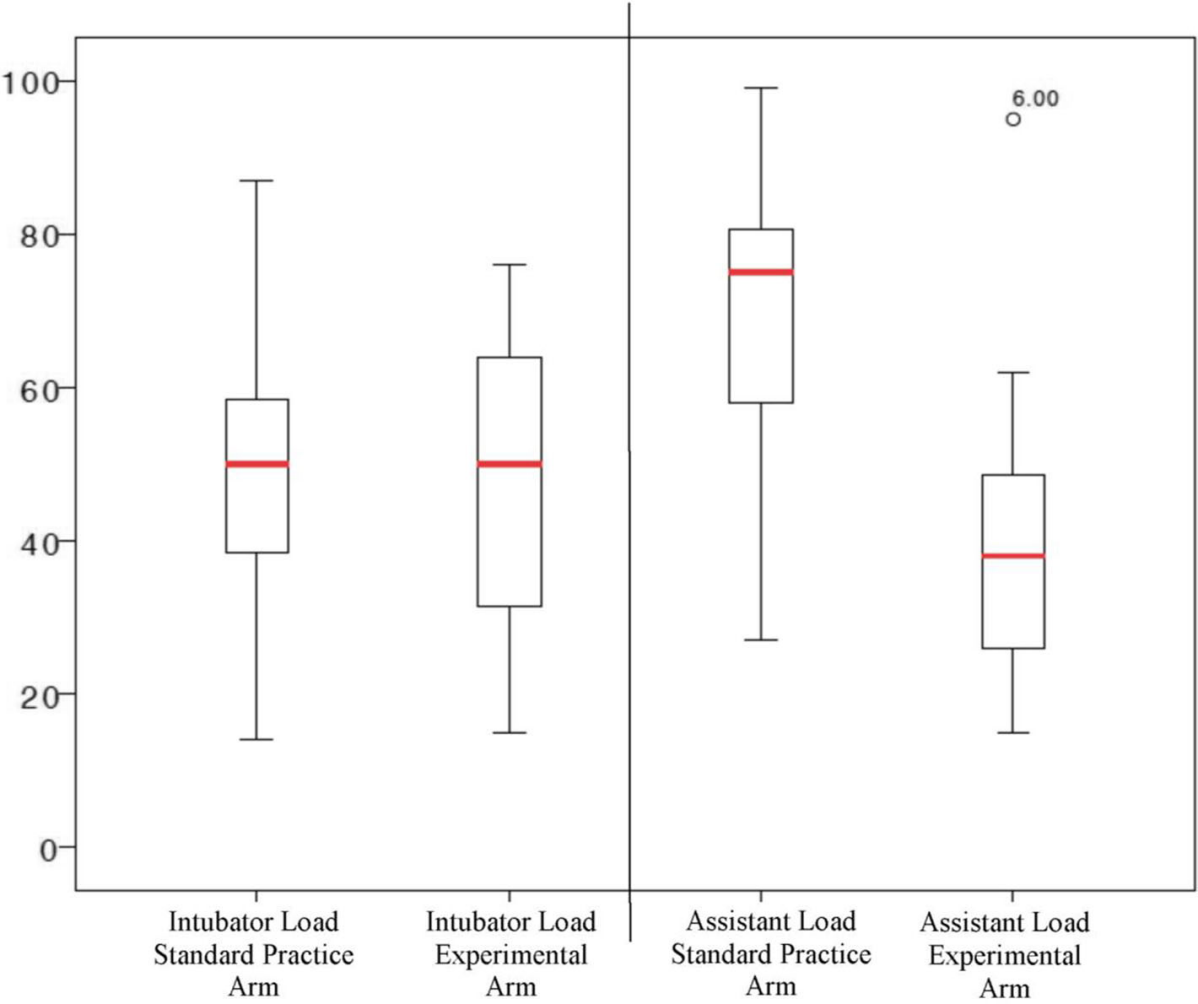
Box-and-whisker plot presenting the individual cognitive load - Standard Practice Arm vs Experimental Arm

Cognitive load of the intubator was similar between groups (49.9 (SD 20.5) mm versus 49.4 (SD 20.5) mm; MD 0.5 (95% CI -16.7 to 17.8) mm; *p* = 0.945) (Students *t*). However, the cognitive load of the intubator assistant was significantly reduced when using the experimental method compared to standard practice: (41.9 (SD 22.4) mm versus 68.7 (SD 24.8) mm; MD 26.8 (95% CI 9.8 to 43.8) mm; *p* = 0.006) (Students *t*) (Fig. 4).

## Discussion

### Key findings

This study demonstrates the effectiveness of a system of pre-preparation of equipment and drugs, together with optimal ergonomic organisation of equipment, for PHEA. Our results show a significant and clinically meaningful reduction in 1) the time it takes to perform the procedure, 2) errors during procedure / or significantly safer performance, and 3) the cognitive load of operators.

When aiming for short scene times (< 30 min) a reduction of 9:14 min is clinically meaningful. Two elements contributed to this: 1) the time to set up the equipment “kit dump” and 2) the time to prepare the required drugs, which accounted for most of the time saved.

Most of the errors and safety-related incidents occurred during the preparation and labelling of drugs on scene. In one incident, the intubator assistant cut a finger while opening an ampoule, highlighting the risk of sustaining a sharps injury when working under pressure. In another, a Rocuronium syringe was not labelled, and then confused for another agent, highlighting the risk of a drug administration error. These errors and safety-related incidents were, however, eliminated by using pre-drawn-up drugs in labelled syringes, resulting in significantly safer operation.

The variance in procedural time was less in the experimental arm, which may suggest an improved workflow. By improving workflow, overall performance was enhanced, and cognitive load was reduced. However, realising where cognitive resilience within a team lies is an important consideration, especially when performing complex, high-risk interventions such as PHEA. In our study, the intubator assistant reported a significantly reduced CL in the experimental method, even lower than that of the intubator. This enables the team to utilise this resilience to their advantage, for example by maintaining the team’s situational awareness “through” the intubator assistant to deliver safe, timely, effective, high quality care as a team.

### Time reduction

The initial resuscitation and evaluation of critically injured or ill patients begins in the pre-hospital environment, and the care that they receive can have a major influence on subsequent outcome [13, 14]. Providing individualised, tailored care based on injury patterns, means that some patients may require specialised care, such as PHEA to optimize their clinical condition prior to transfer [15]. However, these interventions are known to increase time on scene, [2, 16] while the Association of Anaesthetists of Great Britain and Ireland stipulate that “*every effort must be made to keep pre-hospital time to a minimum*”[2]. As a service, we aim to spend time on scene wisely, and minimise time from incident to definitive care. To spend a large proportion of this time preparing for PHEA, while caring for a critically ill or injured patient, is not effective use of time.

Using the concept of “aggregation of marginal gains” [17] and breaking down the intervention (PHEA) into its core components, we identified the preparation phase of a procedure to be critical in determining both the safety of the procedure and the time it takes to perform PHEA. We were then able to demonstrate a significant time reduction in delivering the intervention. It was also clear that most of the preparation for this procedure could be done before the procedure became necessary, i.e. in controlled undisturbed conditions at base rather than on-scene with all the attendant competing demands on our attention and potential for distractions and interruptions. This time saved may be reflected in reducing scene times and time to definitive care. However, performing an intervention more quickly does not automatically mean that it is performed more safely.

### Error reduction

Human error is an important problem in health care, contributing to a high instance of preventable medication errors [18–21]. Preparing drugs is a time-consuming process, requiring precision. Carrying out this critical task, while at the same time treating a critically injured patient in an uncontrolled pre-hospital environment, is far from desirable and inherently prone to error. Using standard practice of PHEA preparation, our study shows that 40% of on-scene time was spent preparing drugs for PHEA, and most of the errors that occurred arose during the preparation of drugs on scene. These included (Table 5): drug labelling errors, omission of labels, poor sharps management and inadvertent “syringe swaps”, all of which can cause serious patient harm [20–22]. For example, routine practice is to prepare Rocuronium (100 mg) in a 10 ml syringe, and Ketamine (200 mg) in a 20 ml syringe. In one observed error, Rocuronium (200 mg) was prepared in a 20 ml syringe, and subsequently incorrectly labelled as Ketamine. This could have resulted in a neuromuscular drug being administered without prior anaesthesia, exposing the patient to harm.

Such incidents are “*almost invariably judged to represent sub-standard care and litigation is almost invariably successful*” [22, 23]. An anaesthetic practice review of 896 drug error reports that a large number of errors involve drugs in similar sized syringes, along with drug preparation errors, which suggest that this is a frequently occurring incident. [24] In a systematic review of drug administration error prevention during anaesthesia, Jensen et al. recommends *“drugs should be presented in prefilled syringes (where possible) rather than ampoules (either for emergency drugs or in general)”* [25]. This is also supported by the Anaesthesia Patient Safety Foundation as part of a *“new paradigm”* to reduce the number of drug related errors, and improve patient safety [26].

Currently, there is wide variation in the way that pre-hospital services prepare drugs for PHEA, including using pharmacy-prepared drugs in pre-filled syringes, teams preparing the drugs at the start of the shift, drawing them up en route to an incident, and drawing them up on scene.

Syringes can be pre-prepared by the service or pharmacy. Individual services would need to consider the associated costs, waste, and shelf life of each method [Additional file 4]. A barrier to pre-prepared drugs maybe the additional cost of pre-prepared drugs or concerns over the risk of drug wastage. The additional cost may, however, be offset by the accompanying reduction in the frequency of errors in preparing intravenous drugs and, more importantly, the iatrogenic harm and human cost of such errors [22, 27]. Furthermore, the magnitude of the time reduction to administer the drugs for PHEA using pre-filled labelled syringes cannot be ignored.

### Reduction of cognitive load

Cognitive load can affect human performance. The effect of human performance on the safe delivery of anaesthesia is widely recognised. Over 40% of adverse outcomes reported to the 4th National Audit Project (NAP4) [4] were attributed to human factors. “*Cognitive resources, though limited, are under conscious control and can be directed from task to task as necessary*” [28]. In the complex and unpredictable pre-hospital environment, the clinician is faced with additional load, beyond that of delivery of the PHEA. The cognitive demands of managing oneself, the team and the environment can exacerbate an escalating workload, risking plan continuation bias and cognitive overload [29]. This can compromise the delivery of safe, effective high quality care [30], as demonstrated in the seminal case of Elaine Bromley, an example of the considerable harm that can result from cognitive overload [31].

There are several ways of reducing cognitive load in critical situations, including the development of strategies such as briefings, flows (workflow patterns), and checklists and limiting the number of critical decisions that need to be made*.* The cognitive burden can potentially be further reduced by standardising the equipment and processes required for the intervention, for example by streamlining packaging or numbering various components sequentially. Such improvements could enhance patient safety by contributing to greater reliability, resilience and situational awareness [22, 4, 32].

There is a recognised relationship between workflow and cognitive load [28] and this can be influenced by the storage and presentation of equipment [33]. If the method of storing and presenting equipment for an intervention is designed to reflect more precisely the series and sequence of steps required for that intervention, the method itself becomes a useful “tool” for reducing the cognitive burden (see Fig. 2).

We hope the findings of this study will support a change in practice from on-scene PHEA drug and equipment preparation to pre-preparation.

We believe that the results of our study are generalisable to any pre-hospital situation where PHEA is being delivered, as factors such as time, safety and cognitive load are the same regardless of the model of pre-hospital care (physician/paramedic, nurse/paramedic, critical care paramedic/paramedic).

The strengths of this study include the realistic simulation of a pre-hospital scenario, allowing unbiased measurement of important aspects of PHEA, which would likely not be possible under real conditions.

Our study has several limitations. This experiment was done in a simulated setting, so the results may not replicate true clinical practice. However, the pre-hospital clinical simulation [Additional file 2] was piloted by clinicians not involved in the study, before the trial recruitment began, to ensure that the simulation was reproducible, straightforward, and that it recreated the clinical practice as closely as possible. It is likely that real pre-hospital cases would be even more complex than those simulated, and would result in even more errors.

The same pre-hospital clinical simulation was used in both methods of the trial which may have introduced exposure bias or training bias. A two-week washout period between the first and the second simulation was implemented to reduce this bias, and clinicians were blind to the outcomes being measured during the simulation. No difference in performance was seen either side of the washout period.

The VAS is used in a wide variety of populations and situations due to its adaptability and ease of use [35]. However, VAS is subjective, and some evidence exists that suggests that it lacks sensitivity and that risks of error exist in some subject groups [36]. We acknowledge that visual analogue scales have not been validated to measure cognitive load in this setting. Nevertheless, we feel that these simple tools are able to provide an unbiased meaningful message, that signal how cognitive resilience could be enhanced during this intervention.

A further limitation of this study is that only 23 of the 24 simulations were included into our analysis as one of the simulations (using standard practice) was incomplete and thus excluded. However, even if the quickest procedure time, across both groups, was input as the missing value, the procedural time using the experimental method remained significantly less.

## Conclusion

Pre-preparation of PHEA drugs, and to a lesser extent the pre-preparation and organisation of PHEA equipment, significantly reduced procedural time and has the potential to reduce on-scene time substantially. In addition, pre-preparation of equipment and drugs resulted in safer performance of PHEA and reduced the cognitive load of the PHEA assistant*.*

## Additional Files

Additional file 1: Research Protocol (PDF 755 kb)

Additional file 2: Pre-hospital clinical simulation (PDF 358 kb)

Additional file 3: Definitions of Prehospital Anaesthesia components (DOCX 77 kb)

Additional file 4: Pharmacy-prepared prefilled syringes cost and shelf life [37, 38] (DOCX 14 kb)

## Abbreviations

CL: Cognitive load
DM: Difference of Medians
EMRS: Emergency Medical Retrieval Service
EtCO_2_: End-tidal capnography
ETT: Tracheal tube
HEMS: Helicopter Emergency Services
ICL: Individual cognitive load
IQR: Interquartile Range
IV: Intravenously
MD: Mean Difference
mm: Milimeters
mm:ss: minutes: seconds
n: Frequency
PHEA: Pre-hospital Emergency Anaesthesia
SAS: Scottish Ambulance Service
ScotSTAR: Scotland’s national Specialist Transport and Retrieval service
SCRAM^®^: Structured CRitical Airway Management
SD: Standard Deviation
SHIL: Scottish Health Innovations
SOP: Standard operating procedure
VAS: Visual analogue score
