# Supplementary material for: Impact of drug and equipment preparation on pre-hospital emergency Anaesthesia (PHEA) procedural time, error rate and cognitive load
Source: Scand J Trauma Resusc Emerg Med. 2018 Sep 21;26:82. doi: 10.1186/s13049-018-0549-3 (PMC6150998; doi:10.1186/s13049-018-0549-3)
Supplement: Supplementary file 1 — Research Protocol (PDF 755 kb) [file 13049_2018_549_MOESM1_ESM.pdf]

PROTOCOL

A Comparison of Error Rate and Time Taken to Perform Pre-Hospital Rapid Sequence Induction of Anaesthesia and Tracheal Intubation Using Prepared and Unprepared Equipment and Drugs

Version 1

08 November 2016

Paul Swinton

Full Title A Comparison of Error Rate and Time Taken to Perform Pre-Hospital Rapid Sequence Induction of Anaesthesia and Tracheal Intubation Using Prepared and Unprepared Equipment and Drugs

Short Title/Acronym Impact of pre-prepared drugs & equipment for pre-hospital RSI.

Sponsor Queen Mary, University of London

Contact person of the above sponsor organisations is: **Sally Burtles**

Director of Research Service & Business Development
Queen Mary Innovation Centre, Lower Ground Floor
5 Walden Street
London
E1 2EF
Phone: 020 7882 7265
Email: sponsorsrep@bartshealth.nhs.uk

REC Reference QMREC1839a

Chief Investigator Dr Zane Perkins (zaneperkins@gmail.com)

Principal Investigator Paul Swinton (pswinton@nhs.net)

Insert as applicable list of Site: Address: Scottish Ambulance Service
ScotSTAR
Air Ambulance Divisional Headquarters
Hanger B
180 Abbotsinch Rd
Paisley, PA3 2RY

Contents Page

2. GLOSSARY OF TERMS AND ABBREVIATIONS

3. SIGNATURE PAGE

4. SUMMARY/SYNOPSIS

5. INTRODUCTION

6. TRIAL OBJECTIVES

7. METHODOLOGY

    Inclusion Criteria

    Exclusion Criteria

    Study Design / Plan – Study Visits

    Study Scheme Diagram

8. STUDY PROCEDURES

    Schedule of Assessment (in Diagrammatic Format)

    End of Study Definition

9. STATISTICAL CONSIDERATIONS

10. ETHICS

11. SAFETY CONSIDERATIONS

12. DATA HANDLING AND RECORD KEEPING

    Confidentiality

    Record Retention and Archiving

13. TECHNIQUES AND TOOLS

14. SAFETY REPORTING

15. MONITORING & AUDITING

16. TRIAL COMMITTEES

17. FINANCE AND FUNDING

18. INDEMNITY

19. DISSEMINATION OF RESEARCH FINDINGS

20. REFERENCES

21. APPENDICES

2. GLOSSARY of Terms and Abbreviations

AE	Adverse Event
AR	Adverse Reaction
ASR	Annual Safety Report
CA	Competent Authority
CI	Chief Investigator
CRF	Case Report Form
CRO	Contract Research Organisation
DMC	Data Monitoring Committee
EC	European Commission
EMRS	Emergency Medical Retrieval Service
GAfREC	Governance Arrangements for NHS Research Ethics Committees
HEMS	Helicopter Emergency Medical Services
ICF	Informed Consent Form
JRMO	Joint Research Management Office
NHS REC	National Health Service Research Ethics Committee
NHS R&D	National Health Service Research & Development
Participant	An individual who takes part in a clinical trial
PI	Principal Investigator
PIS	Participant Information Sheet
PRSI	Pre-hospital rapid sequence induction of anaesthesia and tracheal intubation.
QA	Quality Assurance
QC	Quality Control
RCT	Randomised Controlled Trial
REC	Research Ethics Committee
SAE	Serious Adverse Event
SDV	Source Document Verification
SOP	Standard Operating Procedure
SSA	Site Specific Assessment
TMG	Trial Management Group
TSC	Trial Steering Committee

3. SIGNATURE PAGE

Chief Investigator Agreement

The clinical study as detailed within this research protocol **(Version 1, 08 Nov 16)**, or any subsequent amendments will be conducted in accordance with the Research Governance Framework for Health & Social Care (2005), the World Medical Association Declaration of Helsinki (1996) and the current applicable regulatory requirements and any subsequent amendments of the appropriate regulations.

Chief Investigator Name: Dr Zane Perkins

Chief Investigator Site:

Signature and Date: 12/12/16

Principal Investigator Agreement *(if different from Chief investigator)*

The clinical study as detailed within this research protocol **(Version 1, 08 Nov 16)**, or any subsequent amendments will be conducted in accordance with the Research Governance Framework for Health & Social Care (2005), the World Medical Association Declaration of Helsinki (1996) and the current applicable regulatory requirements and any subsequent amendments of the appropriate regulations.

Principal Investigator Name: Paul Swinton

Principal Investigator Site: ScotSTAR, Scottish Ambulance Service

Signature and Date: 13/12/16

4. SUMMARY/SYNOPSIS

**Short Title**	Impact of pre-prepared drugs & equipment for prehospital RSI.
**Methodology**	Type of study: Randomised control experiment with a cross-over design
**Research Sites**	Scottish Ambulance Service, ScotSTAR
**Objectives / Aims**	Compare the effect on time and safety to set up equipment (kit dump), and place an endotracheal tube during a simulated prehospital scenario. Using different equipment configurations for pre-hospital Rapid Sequence Induction of anaesthesia (RSI). The primary outcome on total intervention time (seconds) and secondary outcomes on safety-related incidents and errors, along with degree of cognitive load, satisfaction, and difficulty scores.
**Number of Participants/Patients**	24 participants forming 12 two person teams (a physician and a paramedic).
**Main Inclusion Criteria**	**Retrieval Physicians:**<br>• Currently working with the adult retrieval service of ScotSTAR<br>- Emergency Medical Retrieval Service (EMRS).<br>• Greater than 6 months' experience as a consultant physician with EMRS.<br>• Assessed by EMRS to be competent and current at preparing a kit dump for pre-hospital RSI.<br><br>**Helicopter emergency services (HEMS) Paramedics:**<br>• Currently working for the Scottish Ambulance Service air ambulance division alongside EMRS.<br>• Greater than 6 months' experience as a HEMS paramedic.<br>• Minimum of 8 years' frontline ambulance experience.<br>• Completed pre-hospital anaesthesia course[1] or equivalent<br>• Assessed by EMRS to be competent and current at preparing a kit dump for pre-hospital RSI.<br>REFERENCES:<br>1. Pre-hospital anaesthesia course. GNAAS. https://www.greatnorthairambulance.co.uk/pha/Accessed January 25, 2018.
**Statistical Methodology and Analysis (if applicable)**	Distribution of data will be assessed using normal quartile plots (or an appropriate test).<br>However, we are aware that each of these statistical tests have their own

	limitations.<br><br>**If data is parametric then:**<br>Catagorical data compared using Chi^2, continuous data compared with Student T.<br><br>**If non-parametric then:**<br>Catagorical data compared with Fischer Exact, continuous data compared with Wilcoxon matched-pair signed-rank test.<br><br>**Primary outcome** will compare the mean total intervention time to completion of the process by standard practice vs experimental method. p-value will be set at <0.05 for statistical significance.<br><br>**Secondary outcomes** of component times (table 1) will compare the mean time to completion of each component by standard practice vs experimental method.<br><br>**Secondary outcome** will be measured using 100mm visual analog score, comparing the mean satisfaction, degree of cognitive load by standard practice vs experimental method.
**Proposed Start Date**	01 December 2016
**Proposed End Date**	01 July 2017
**Study Duration**	7 Months

5. INTRODUCTION

Rapid Sequence Induction of anaesthesia (RSI) in any environment is associated with significant risks including hypoxia, hypotension, or failure to secure an airway. Most of these complications are predictable, and the risk can be greatly reduced with appropriate preparation. In controlled environments, for example the induction of anaesthesia for elective surgery, these risks are extremely low. However, in uncontrolled environments these risks are more likely to increase.

A potential source of error in pre-hospital RSI is the preparation of the required equipment and drugs. Pre-hospital time is an essential factor in the initial care of critically injured/ill patients, and is directly related to mortality. Time saving interventions are therefore beneficial.

One of the principal aims of pre-hospital care is to balance the need for appropriate intervention with the need to transport patients to definitive care.

This study will evaluate the effectiveness of a new method of working, having all necessary equipment and drugs prepared and organised prior to the procedure being required, to investigate whether the improved pre-preparation will make this process faster, while still resulting in the safe placement of endotracheal tubes.

6. TRIAL OBJECTIVES

Research Questions:

- Does pre-preparing drugs and equipment for pre-hospital rapid sequence induction of anaesthesia and tracheal intubation (PRSI) impact the time it takes to safely place an endotracheal tube?

- Does pre-preparing drugs and equipment for pre-hospital RSI impact the error rate (safety)?

Objective:

Compare the effect on time and safety to set up equipment (kit dump) and place an endotracheal tube during a simulated prehospital scenario. Using different equipment configurations for pre-hospital Rapid Sequence Induction of anaesthesia (RSI). The primary outcome on total intervention time (seconds) and secondary outcomes on safety related incidents and errors, along with degree of cognitive load, satisfaction, and difficulty scores.

Definitions / Endpoints

- Primary

• Total intervention time (seconds) - defined in this study, from the decision to preform RSI to confirming placement of endotracheal tube (breathing tube) with the measure of end-tidal capnography (concentration of carbon dioxide at the end of an exhaled breath).

- Secondary

• Component times (seconds) - being the time taken for each component of the process (table 1) to include preparation of airway equipment and drug preparation, checklist, drug administration, onset time, tracheal intubation.

• Safety related incidents

- Procedural errors, defined as error in the preparation or use of medications or equipment with the potential to result in harm an unintended/unexpected incident, which led, or could have led to harm. Errors will be counted and classified according to table 2.

- Procedural lapse, defined as a failure to execute an action due to lapse in memory and a routine behaviour being omitted. Lapse will be counted and classified according to table 2

• Degree of cognitive load - Subjective estimate, using a visual analogue scale, of the amount of cognitive work the procedure entailed. Cognitive load will be defined for the participant as the amount of cognitive work/energy required to complete the procedure, including the level of judgements/decisions needing to be made.

7. METHODOLOGY

Inclusion Criteria

Eligible participants are experts in prehospital care, and perform RSI as part of their normal working practice. They will be retrieval consultant physicians recruited from Scotland's National Retrieval Service (ScotSTAR) and Helicopter Emergency Services (HEMS) Paramedics employed by the Scottish Ambulance Service.

Each participant will be emailed an invitation that includes an information sheet about the nature of the study; consenting participants are then enrolled.

Participants will be randomly assigned into 12 teams of two (a physician and a paramedic) using computer randomisation. Each team will be asked to perform an RSI on a mannequin in a simulated pre-hospital setting.

The trial will comprise of a traditional set-up of a drug bag containing all the required drug vials, syringes and labels to prepare for a pre-hospital RSI, and a conventional airway bag holding all the required airway equipment, versus the new experimental set-up using a new method of working, having all necessary equipment and drugs prepared and organised prior to the procedure being required.

Each team will use both set-ups sequentially during the two scenarios, their starting process (standard practice or experimental method) being decided randomly using computer batch randomisation. To reduce exposure bias and training artefact there will be a minimum period of two weeks between the first and the second scenario. The allocation will be conveyed to the participant in sequential-numbered opaque envelopes, and undertaken independently for each participant group to ensure an adequate number of participants in each arm.

Before commencing their assigned arm of the study each team will be briefed on what is needed and provided as much time as required to prepare, and ask questions. A picture of a kit dump will be used to indicate what is required and standardize the briefing.

The time taken to complete the process will be measured and the difference between the two set-ups compared, along with safety, errors, and degree of cognitive load, satisfaction, and difficulty scores received from the participants. It is anticipated that this process will

take approximately 1 hour of each participants' time. To accurately measure the outcomes, filming will be used, solely for review by the investigators, and then destroyed.

The investigator will record:

- Total intervention time (seconds) - defined in this study, from the decision to preform RSI to confirming placement of endotracheal tube (breathing tube) with the measure of end-tidal capnography (concentration of carbon dioxide at the end of an exhaled breath).

- Component times (seconds) - being the time taken for each component of the process (table 1) to include preparation of airway equipment and drug preparation, checklist, drug administration, onset time, tracheal intubation.

- Procedural errors, defined as error in the preparation or use of medications or equipment with the potential to result in harm an unintended/unexpected incident, which led, or could have led to harm. Errors will be counted and classified according to table 2.

- Procedural lapse, defined as a failure to execute an action due to lapse in memory and a routine behaviour being omitted. Lapse will be counted and classified according to table 2.

- The degree of individual cognitive load (ICL), defined as the amount of cognitive work/energy required by the participant to complete the procedure, including the level of judgements/decisions needing to be made will be measured.

Table 1 - *definitions of components of time*

	Description	Definition
	Total Intervention time	Total time taken, from decision to RSI to the measure of $EtCO_2$.
Component times	Equipment Preparation	Total time taken to setup the airway equipment, from touching the airway bag to completing the equipment setup.
	Drug Preparation	Total time to prepare the drugs, from touching the drug bag to completing the preparation of the drugs.
	Checklist	Total time to complete the checklist, from the point of starting the checklist to completing the last sentence of the checklist.
	Drug Administration	Total time taken to administer the drugs, from picking up the syringe to complete administration of Alfentanil, Ketamine and Rocuronium.
	Drug Onset Time	The time it takes for drugs to reach maximal effect, for optimal intubation conditions (60sec), from the administration of Rocuronium to the decision to attempt intubation.
	Tracheal Intubation	Placement of an endotracheal tube into the trachea, under direct laryngoscopy, confirmed by visualising it pass through the vocal cords, by auscultation and by the measure of quantitative $EtCO_2$

Table 2 Error Classifications

Classification	Definitions	Examples
Error	Procedural error in the preparation or use of medications or equipment with the potential to result in harm	**Medication:**<br>o Syringe containing anaesthesia medication labelled incorrectly or not labelled<br>o Incorrect medication administered<br>o Incorrect dose administered<br><br>**Equipment:**<br>o Sharps injury<br>o Procedure performed not in accordance with SOP (i.e. checklist not used, bougie not used)
Lapse	A failure to execute an action due to lapse in memory and a routine behaviour being omitted.	**Medication preparation:**<br>o Same needle used to draw up multiple medications<br>o No syringe cap<br>o Unsafe sharps management<br><br>**Equipment preparation:**<br>o Cuff of endotracheal tube not checked<br>o Laryngoscope bulb operation not checked<br>o No bougie

Exclusion Criteria

Clinicians who have less than 6 months experience working with or alongside the adult retrieval service (EMRS).

Clinicians who have not been assessed by EMRS to be competent and current at preparing a kit dump for pre-hospital Rapid Sequence Induction of anesthesia (PRSI).

Study Design / Plan – Study Visits

Eligible participants are experts in prehospital care, and perform RSI as part of their normal working practice. They will be retrieval physicians recruited from Scotland's National Retrieval Service (ScotSTAR) and Helicopter Emergency Services (HEMS) Paramedics employed by the Scottish Ambulance Service.

Each participant will be emailed an invitation that includes an information sheet about the nature of the study; consenting participants are then enrolled.

Participants will be randomly assigned into 12 teams of two (a physician, and a paramedic) using computer randomisation.

Study Scheme Diagram

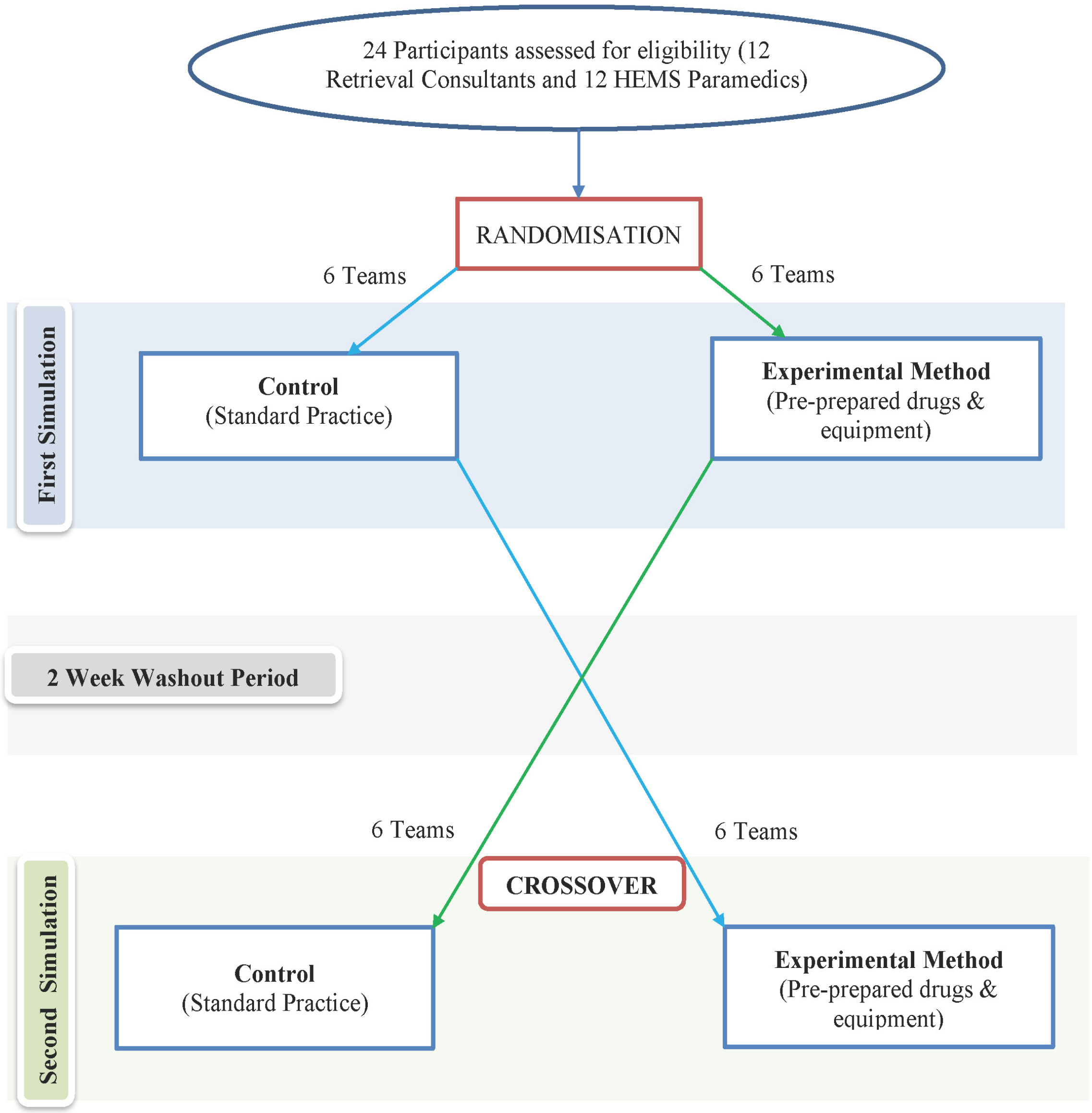

8. STUDY PROCEDURES

Informed Consent Procedures and Enrollment

All the voluntary participants are consenting adults, each of whom will firstly have received an information sheet pertaining to this study. In accordance with the Data Protection Act 1998. We will follow ethical and legal practice, all information which is collected about the participant during the research will be kept strictly confidential.

Participants will be allocated a unique study reference number and all data collected will be stored under this number, including questionnaire responses. Participants will also be informed, that filming will be used to accurately measure the outcomes which will only be reviewed by the investigators, after which it will be destroyed. Identifiable electronic data will be stored on an NHS computer in a password protected domain. This information will only be accessible to the researcher.

Each of the participants will be briefed on what they will be expected to do during the study, and advised how their anonymised data will be used. The participant is free to withdraw at any stage, and should they decide to withdraw all their data will be destroyed and will not be included in any subsequent publication.

They will be provided time to ask any questions and once they are happy to proceed the participant will sign a consent form prior to starting.

Randomization Procedures

Each participant (physician and paramedic) will be randomly appointed using computer randomisation into a two-person (physician, paramedic) team.

In addition, computer batch randomisation will be undertaken to establish the first process which would be undertaken by the team (standard practice or experimental arm). The allocation will then be conveyed to the participants by means of sequential numbered opaque envelopes. To ensure adequate inclusion of each team to both arms of the study, this process will be undertaken independently for each participant team.

Schedule of Assessment (in Diagrammatic Format)

Simulation	Total Intervention Time	Component time	Safety related incidents: Errors	Safety related incidents: Lapse	Cognitive load	Difficulty	Satisfaction
First	x	x	x	x	x	x	x
Second	x	x	x	x	x	x	x
Measure	Time in seconds		Number of events		100mm analogue scale		

End of Study Definition

Once all the teams have completed both arms of the study.

9. STATISTICAL CONSIDERATIONS

Sample Size for the study.

- Total UK sample size: 24
- Total international sample size (including UK): 24
- Total in European Economic Area: 24

- *Sample size for the study.*

24 participants forming 12 two person teams.

Statistical explanation for sample size for the study.

- Sample size was calculated using data from observation of prior EMRS practice: in ten consecutive standard PRSI's, measuring 16:03 to 24:28 minutes: seconds, the mean procedural time was 20:03 minutes: seconds and standard deviation of 3:26 minutes: seconds.

- A 20% reduction in procedural time would be considered clinically significant.

- From this information, the sample size needed for the paired t-test to have a 90% chance of detecting a difference in means of four minutes at a level of significance of 5% (two-sided), is 11 samples. To allow for any exclusions the sample size is adjusted to twelve simulations in each arm.

- *Method of Analysis*

Distribution of data will be assessed using normal quartile plots (or an appropriate test). However, we are aware that each of these statistical tests have their own limitations.

If data is parametric then:

Categorical data compared using Chi^2, continuous data compared with Student-T.

If non-parametric then:

Categorical data compared with Fischer Exact, continuous data compared with Wilcoxon matched-pair signed-rank test.

- Primary Outcome

• will compare the mean total intervention time to completion of the process by standard practice vs experimental method. p-value will be set at <0.05 for statistical significance.

- Secondary outcomes

- Component times (seconds) - being the time taken for each component of the process (table 1) by standard practice vs experimental method.

• Safety related incidents

    - Procedural errors, will be counted and classified according to table 2 by standard practice vs experimental method.

    - Procedural lapse, will be counted and classified according to table 2 by standard practice vs experimental method.

• Degree of cognitive load - will be measured using 100mm visual analog score, comparing the mean satisfaction, degree of cognitive load by standard practice vs experimental method.

- Primary Endpoint

- Total intervention time (minutes: seconds) - defined in this study as starting at the decision to perform PRSI and ending when correct ETT position, confirmed with the

facilitator turning on the $EtCO_2$ simulation software, in response to visualising chest inflation.

- *Secondary Endpoint*

- Component times (seconds) - Time taken to complete each of the components of the procedure are presented in table 1.

- Procedural errors, defined as an unintended/unexpected incident, which led, or could have led to harm. Errors were counted and classified according to table 2.

- Individual cognitive load (ICL), defined as the amount of cognitive work/energy required by the participant to complete the procedure, including the level of judgements/decisions needing to be made will be measured. ICL will be measured using visual analogue score (VAS). At the end of each simulation, participants will be asked to record their perceived degree of cognitive load during PRSI on a standard 100-mm line (0-mm representing no cognitive load and 100-mm representing maximal cognitive load) comparing the means with the process by standard practice vs experimental method.

10. ETHICS

The Principal Investigator will ensure that the study will be carried out in accordance with the ethical principles in the Research Governance Framework for Health and Social Care, Second Edition, 2005 and its subsequent amendments as applicable and applicable legal and regulatory requirements.

This study is considered exempt from NHS Research Ethics in Scotland. In addition, details were submitted to Queen Mary University of London Research Ethics Committee, who have deemed that this research does not present any ethical concerns and is extremely low risk and thus does not require the scrutiny of the full Research Ethics Committee (QMREC1839a).

All the voluntary participants are consenting adults, each of whom will firstly have received an information sheet pertaining to this study. In accordance with the Data Protection Act 1998. We will follow ethical and legal practice, all information which is collected about the participant during the research will be kept strictly confidential.

Participants will be allocated a unique study reference number and all data collected will be stored under this number, including questionnaire responses. Participants will also be informed, that filming will be used to accurately measure the outcomes which will only be reviewed by the investigators, after which it will be destroyed. Identifiable electronic data will be stored on an NHS computer in a password protected domain. This information will only be accessible to the researcher.

Each participant will be briefed on what they will be expected to do during the study, and advised how their anonymised data will be used. They will be provided time to ask any questions, once satisfied to proceed they will sign a consent form prior to starting. The participant is free to withdraw at any stage, and should they decide to withdraw all their data will be destroyed and will not be included in any subsequent publication.

Include details of any conflicts of interest.

The airway bag (SCRAM® bag) which is used in the experimental group of this study was designed and developed by Paul Swinton and Neil Sinclair both of whom work for the Scottish Ambulance Service. The SCRAM® bag is now a commercially available product and as such Paul Swinton and Neil Sinclair receive a royalty. The Scottish Ambulance Service holds intellectual property rights to the SCRAM® bag.

Scottish Health Innovations Ltd (SHIL) is a publicly owned private company limited by guarantee, wholly owned by Scottish Ministers and two health boards. SHIL was set up in 2002 by NHS Scotland and Scottish Enterprise to support innovation in the NHS. SHIL works in partnership with NHS Scotland to support and develop innovative solutions which address a clearly identified healthcare needs; with the proposed innovations being generated by NHS healthcare professionals. By developing these ideas, SHIL creates new products and technologies that aim to improve patient care and generate income for NHS Scotland.

• SHIL have helped us develop an idea into a product, that is now commercially available. Where 1/3 of the proceeds go to the employer (SAS), 1/3 is equally divided between the inventors and the remaining goes to the manufacturer.

• SHIL have also funded the cost (£872) of the consumables for this study.

11. SAFETY CONSIDERATIONS:

During the simulated preparation of the kit dump for emergency prehospital anaesthesia, the participants will be expected to draw up drugs, which is part of their everyday practice and these experienced clinicians are aware of safe sharps management. Nevertheless, all participants will be reminded to adhere to the safe practice and working with sharps. However, in the event a sharps injury is sustained a first aid kit will be available.

12. DATA HANDLING AND RECORD KEEPING:

- *Confidentiality*

In accordance with the Data Protection Act 1998. We will follow ethical and legal practice, all information which is collected about the participant during the course of the research will be kept strictly confidential and managed in accordance with the Data Protection Act, NHS Caldecott Principles, The Research Governance Framework for Health and Social Care, and the conditions of Research Ethics Committee Approval. Participants will be allocated a unique study reference number and all data collected will be stored under this number, including questionnaire responses. Identifiable electronic data will be stored on an NHS computer in a password protected domain. This information will only be accessible to the researcher.

The lever arch file containing the enrolment forms and raw data will be secured in a locked filing cabinet at the ScotSTAR base (address below) where only the principle investigator has access to.

To accurately measure the outcomes, filming will be used and will be reviewed by investigators to accurately record the timings of elements of the procedure, after which it will be destroyed.

- *Record Retention and Archiving*

The data generated by the study will be stored for 5 years on an NHS computer in a password protected domain.

The lever arch file containing the enrolment forms and raw data will be secured in a filing cabinet at the ScotSTAR base (address below) where only the chief investigator has access to.

No recorded film will be retained. In accordance with the data protection act 1998. At the end of the study all personal enrolment data will be securely destroyed.

Address: Scottish Ambulance Service | Air Ambulance Divisional Headquarters| Hanger B | 180 Abbotsinch Rd | Paisley | PA3 2RY.

13. TECHNIQUES AND TOOLS

Techniques and interventions

In both arms of the experiment, the clinical team will be asked to perform performed PRSI on a mannequin, *presented within a realistic pre-hospital clinical simulation* (Appendix 3). This included the decision to RSI, and the performance of the procedure according to the services (SOP).

Tools

Following the simulation, participants will complete a questionnaire (100mm analogue scale) measuring cognitive load, satisfaction, and difficulty.

14. SAFETY REPORTING

Adverse Events (AE)

An AE is any untoward medical occurrence in a subject to whom a medicinal product has been administered, including occurrences which are not necessarily caused by or related to that product. An AE can therefore be any unfavourable and unintended sign (including an abnormal laboratory finding), symptom or disease temporarily associated with study activities.

Notification and reporting Adverse Events or Reactions

If the AE is not defined as SERIOUS, the AE is recorded in the study file and the participant is followed up by the research team. The AE is documented in the participants' medical notes (where appropriate) and the CRF.

Serious Adverse Event (SAE)

In other research other than CTIMPs, a serious adverse event (SAE) is defined as an untoward occurrence that:

(a) results in death;

(b) is life-threatening;

(c) requires hospitalisation or prolongation of existing hospitalisation;

(d) results in persistent or significant disability or incapacity;

(e) consists of a congenital anomaly or birth defect; or

(f) is otherwise considered medically significant by the investigator.

An SAE occurring to a research participant should be reported to the main REC where in the opinion of the Chief Investigator the event was:

- Related – that is, it resulted from administration of any of the research procedures, and
- Unexpected – that is, the type of event is not listed in the protocol as an expected occurrence.

Notification and Reporting of Serious Adverse Events

Serious Adverse Event (SAEs) that are considered to be 'related' and 'unexpected' are to be reported to the sponsor within 24 hours of learning of the event and to the Main REC within 15 days in line with the required timeframe.

Urgent Safety Measures

The CI may take urgent safety measures to ensure the safety and protection of the clinical trial subjects from any immediate hazard to their health and safety. The measures should be taken immediately. In this instance, the approval of the REC prior to implementing these safety measures is not required. However, it is the responsibility of the CI to inform the sponsor and Main Research Ethics Committee (via telephone) of this event immediately.

The CI has an obligation to inform both the Main REC in writing within 3 days, in the form of a substantial amendment. The sponsor (Joint Research Management Office [JRMO]) must be sent a copy of the correspondence with regards to this matter.

Annual Safety Reporting

The CI will send the Annual Progress Report to the main REC using the NRES template (the anniversary date is the date on the MREC "favourable opinion" letter from the MREC) and to the sponsor.

Overview of the Safety Reporting responsibilities

The CI/PI has the overall pharmacovigilance oversight responsibility. The CI/PI has a duty to ensure that safety monitoring and reporting is conducted in accordance with the sponsor's requirements.

Safety will be monitored in accordance with standard NHS health and safety procedures. Safety will be reviewed on a case by case basis to establish if any interventions are required. In view of the small sample size and service
evaluation methodology for this study, interim analysis of efficacy is not indicated.

What are the criteria for electively stopping the trial or other research prematurely?

Clear evidence of participant risk or an instance of significant injury to a participant will prompt a safety stop and review. Such a stop would be reviewed at local and QMUL level with regard to continuing the study with appropriate modifications to protect the participants with a view to achieving the primary outcome measures. Failing this the trial would be terminated by the PI at the sole site.

15. MONITORING & AUDITING

This is a single site study which will be overseen by the PI and team who will be responsible for conduct of the research. Overall monitoring will be undertaken by the QMUL with local oversight from the ScotSTAR R&D group.

> Definition:
> "A systematic and independent examination of trial related activities and documents to determine whether the evaluated trial related activities were conducted, and the data were recorded, analysed and accurately reported according to the protocol, sponsor's standard operating procedures (SOPs), Good Clinical Practice (GCP), and the applicable regulatory requirement(s)."

A study may be identified for audit by any method listed below:

1. A project may be identified via the risk assessment process.

2. An individual investigator or department may request an audit.

3. A project may be identified via an allegation of research misconduct or fraud or a suspected breach of regulations.

4. Projects may be selected at random. The Department of Health states that Trusts should be auditing a minimum of 10% of all research projects.

5. Projects may be randomly selected for audit by an external organisation.

Internal audits may be conducted by a sponsor's or funder representative.

16. TRIAL COMMITTEES

There are no trial committees.

17. FINANCE AND FUNDING

The airway bag (SCRAM® bag) which is used in the experimental group of this study was designed and developed by Paul Swinton and Neil Sinclair both of whom work for the Scottish Ambulance Service. The Scottish Ambulance Service holds intellectual property rights to the SCRAM® bag.

Scottish Health Innovations Ltd (SHIL) is a publicly owned private company limited by guarantee, wholly owned by Scottish Ministers and two health boards. SHIL was set up in 2002 by NHS Scotland and Scottish Enterprise to support innovation in the NHS. SHIL works in partnership with NHS Scotland to support and develop innovative solutions which address a clearly identified healthcare needs; with the proposed innovations being generated by NHS healthcare professionals. By developing these ideas, SHIL creates new products and technologies that aim to improve patient care and generate income for NHS Scotland.

• SHIL have helped us develop an idea into a product, that is now commercially available. Where 1/3 of the proceeds go to the employer (SAS), 1/3 is equally divided between the inventors and the remaining goes to the manufacturer.

• SHIL have also funded the cost (£872) of the consumables for this study.

18. INDEMNITY

Queen Mary University of London

19. DISSEMINATION OF RESEARCH FINDINGS:

This study would be relevant to clinicians in the following fields:

- Airway management
- Pre-hospital medicine
- Aeromedical services
- Retrieval services
- Trauma services
- Emergency Medicine

The following journal may be considered for publication

Journal	**Target Audience**	**Impact Factor**
Annals of Emergency Medicine	Emergency Medicine	5.532
British Journal of Anaesthesia	Anaesthesia & Airway Management	5.616
Resuscitation	Anaesthesia & Airway Management	5.414
Anaesthesia	Anaesthesia & Airway Management	4.741
Pre-Hospital Emergency Care	Pre-hospital Medicine	2.2
Emergency Medicine Journal	Emergency Medicine	1.836

20. REFERENCES

AMA/Vancouver format using Endnote referencing management software.

21. APPENDICIES

Are stand-alone documents:

1) Simulation (Appendix S2)
