# Supplementary material for: Impact of drug and equipment preparation on pre-hospital emergency Anaesthesia (PHEA) procedural time, error rate and cognitive load
Source: Scand J Trauma Resusc Emerg Med. 2018 Sep 21;26:82. doi: 10.1186/s13049-018-0549-3 (PMC6150998; doi:10.1186/s13049-018-0549-3)
Supplement: Supplementary file 2 — Pre-hospital clinical simulation (PDF 358 kb) [file 13049_2018_549_MOESM2_ESM.pdf]

MOULAGE

Impact of pre-prepared drugs & equipment for pre-hospital RSI. v1

Equipment needed

☐ Pre-hospital response bags	☐ Ambulance trolley and Scoop	☐ Suction unit
☐ MRX	☐ 1x Sharps Box	☐ 2x Oxygen ZD and non -rebreather mask
☐ 1x Manikin	☐ Trial Paper work	☐ 1 Inco pad

Preparation

- ☐ Set up filming equipment, Consent forms, Randomisation
- ☐ Manikin on trolley and scoop with Inco pad under manikin, and covered with thermal blanket
- ☐ O_2 attached to patient and O_2 cylinder, C collar and Pelvic binder in place secured and in place.
- ☐ 2 large bore iv cannula secured and in place with 1 bag of fluid 0.9%NaCl up (not running)
- ☐ Suction unit near trolley, monitoring equip attached and vitals detailed below
- ☐ Pre-brief: Open appropriate drug and equipment bags, **participants can refer to the EMRS SOP and picture of kit dump during this time only.**
- ☐ **Provide a copy of the participant information sheet and the Scottish Ambulance Service SOP Emergency Anaesthesia version 5.**
- ☐ **Complete consent and team log.**

Specific

Set 1 (Control Arm)

Drug bag:

- ☐ Vials - Alfentanil, Ketamine, Rocuronium, Morphine, Midazolam
- ☐ Syringes: 1x2ml, 1x5ml, 2x10ml, 1x20ml and drawing up needles
- ☐ Labels: Alfentanil, Ketamine, Rocuronium, Morphine, Midazolam
- ☐ Pen/marker:
- ☐ **Conventional Airway bag** - Stocked per picture.

Set 2 (Experimental Arm)

Drug bag -

- ☐ Prefilled - Alfentanil, Ketamine, Rocuronium, Morphine, Midazolam **(Correctly labelled and filled with water)**
- ☐ **SCRAM bag -** stocked

MOULAGE

Impact of pre-prepared drugs & equipment for pre-hospital RSI. v1

1

- ☐ **Provide candidate time to familiarize themselves with the equipment and ask questions NOTE they should be blinded to the outcomes.**
- ☐ **Drugs and fluids can be administered to the manikin. This simulation requires the same speed and accurately that you demand of yourself in real life in accordance with the SOP.**

Background

You are working together on helimed 5 when at 16:00 a HEMS activation to Aberfoyle comes through.
Note: Nearest hospital with neurosurgery to this location is QEUH (by road 45min by air 10min)

Description:

- A pedestrian crossing the road near Aberfoyle struck by a light motor vehicle.
- He was apparently thrown 6ft into the air. The telephone caller described the patient as having a head injury, is very distressed and confused.
- No further information is available yet.
- An ambulance has also been dispatched however they are 10min away
- Your everything you might need for your primary survey and initial stabilization of the patient.

On scene:

The police have stopped traffic on scene, after landing, an ambulance crew gives you a handover: Male age 40yrs struck by a light vehicle there is a dent in the bonnet and a shattered windscreen. He has a head injury, initially confused but now responds to Pain. Due to mechanism, we have bound his pelvis, and have 2x IV access points. He is on O2 and that's as far as we have got.

On assessment:
Injury: Large haemotoma to the occiput with blood from ear (note to researcher: this is an isolated head injury)

2

80KG Male

C: No catastrophic haemorrhage
Police ask: is this life threatening or life changing

3

A:	B:	C:	D:
Pt is snoring<br><br>Jaw thrust opens the airway but needs to be maintained	No evidence of chest injury<br><br>RR 10 (Reg)<br><br>SaO2 89<br><br>(Note: amb crew cannot help)<br><br>**After jaw thrust**<br>RR ↑ 20<br><br>**With O^2**<br>↑SaO2 98%<br><br>E_TCO_2: 5	Pulse 52,<br><br>BP 158/100<br><br>**(No evidence of haemorrhagic shock)**<br><br>Abdo: soft<br><br>No evidence pelvic #<br><br>No long bone fractures	AVPU: **P**<br><br>**GCS: 8 / 15**<br><br>**E2** - opens eyes to pain<br><br>**V2** - incomprehensible groans<br><br>**M4** - withdraws from pain<br><br>Pupils: Equal<br>**L** Size 2 (Response Sluggish)<br><br>**R** Size 2 (Response Sluggish)

4

Pre-oxygenation

5

Check List

Straight Forward

7

Drugs*	Dose*
Alfentanyl	1mg-2mg
Ketamine 2mg/kg	160mg
Rocuronium 1 - 1.2mg/kg	80mg - 96mg

*** Within the described dose range of the SOP**

6

Pre Intubation

HR 60

BP160/95

SaO2 98%

8 **Grade 1 View**

9 **Post Intubation**

HR 60   BP165/95   SaO2 98% E_TCO_2: 4.5

10 **MOULAGE ENDS. Once ETT secured and correct placement confirmed WITH $ETCO^2$**

After scenario check syringes, note any label errors and dose that was administrated on the team log

IRAS Project ID: 216904
