# Supplementary material for: Impact of drug and equipment preparation on pre-hospital emergency Anaesthesia (PHEA) procedural time, error rate and cognitive load
Source: Scand J Trauma Resusc Emerg Med. 2018 Sep 21;26:82. doi: 10.1186/s13049-018-0549-3 (PMC6150998; doi:10.1186/s13049-018-0549-3)
Supplement: Supplementary file 3 — Definitions of Prehospital Anaesthesia components (DOCX 77 kb) [file 13049_2018_549_MOESM3_ESM.docx]

| Additional file 3 - Definitions of Prehospital Anaesthesia components | | |
| --- | --- | --- |
| **Description** | **Definition** |  |
| Total Intervention Time | Total time taken, from decision to preform Pre-hospital Emergency Anaesthesia (PHEA) to the measure of EtCO_2._ |  |
| Equipment Preparation | Total time taken to setup the airway equipment, from touching the airway bag to completing the equipment setup. |  |
| Drug Preparation | Total time to prepare the drugs, from touching the drug bag to completing the preparation of the drugs. |  |
| Checklist | Total time to complete the checklist, from the point of starting the checklist to completing the last sentence of the checklist. |  |
| Drug Administration | Total time taken to administer the drugs, from picking up the syringe to complete administration of Alfentanil, Ketamine and Rocuronium. |  |
| Drug Onset Time | The time it takes for drugs to reach maximal effect, for optimal intubation conditions (60sec), from the administration of Rocuronium to the decision to attempt intubation. |  |
| Tracheal Intubation | Placement of an endotracheal tube into the trachea, under direct laryngoscopy, confirmed by visualising it pass through the vocal cords, by auscultation and by the measure of quantitative EtCO_2_ |  |
