# Supplementary material for: Impact of drug and equipment preparation on pre-hospital emergency Anaesthesia (PHEA) procedural time, error rate and cognitive load
Source: Scand J Trauma Resusc Emerg Med. 2018 Sep 21;26:82. doi: 10.1186/s13049-018-0549-3 (PMC6150998; doi:10.1186/s13049-018-0549-3)
Supplement: Supplementary file 4 — Pharmacy-prepared prefilled syringes cost and shelf life [37, 38] (DOCX 14 kb) [file 13049_2018_549_MOESM4_ESM.docx]

| **Additional file 4**Pharmacy-prepared prefilled syringes cost and shelf life [37] | | | |
| --- | --- | --- | --- |
| Drug | Cost / Unit | Shelf life once in prefilled syringe | Cost / Ampoule |
| Alfentanil | £12.50 | Two months | 2ml ampoule = 44p |
| Ketamine | £19 | Two months | 20ml ampoule = £5.06 |
| Rocuronium | *-* | 24hrs^a^ | 5ml ampoule costs £1.20 |
| Morphine | £8.20 | Two- six months | 10p / 10mg ampoule +  5p for the Sodium Chloride to dilute |
| Midazolam | £7.50 | two months | 18p / 5mg ampoule.  36p for 10mg dose |
| Suxamethonium | £12.50 | Two months | £2.88 / ampoule.  £5.76 for 200mg dose |
| ^a^ Guidance on intravenous infusions [38] | | | |
